# Gender inequalities and household fuel choice in India

**DOI:** 10.1016/j.jclepro.2020.121487

**Published:** 2020-08-20

**Authors:** Pallavi Choudhuri, Sonalde Desai

**Affiliations:** a National Council of Applied Economic Research, New Delhi, India; b University of Maryland College Park, USA

## Abstract

The use of solid cooking fuels—wood, straw, crop residue, and cow-dung cakes—is associated with higher levels of environmental pollution and health burden. However, even in an era when incomes have grown and poverty has declined, the proportion of Indian households using clean cooking fuels such as kerosene or Liquefied Petroleum Gas (LPG) has increased only slightly. Even among the wealthiest quintile, only about 40 percent of the households rely solely on clean fuel. Since the chores of cooking and collection of fuel remain primarily the domain of women, we argue that intra-household gender inequalities play an important role in shaping the household decision to invest in clean fuel. Analyses using data from the India Human Development Survey (IHDS), a panel survey of over 41,000 households conducted in two waves in 2004-05 and 2011–12, respectively, show that women’s access to salaried work and control over household expenditure decisions is associated with the use of clean fuel.

## 1 Introduction

The use of solid cooking fuel is associated with higher levels of environmental pollution and health burden (Balakrishnan et al., 2011; Chafe Zoë et al., 2014; Lacey et al., 2017). According to the Global Burden of Disease Studies (GBD) exposures to ambient and household fine-particulate matter (PM_2.5_) together are among the single largest single causes of premature mortality in India. Using GBD 2017 data, Balakrishnan et al. (2019) indicate that of the 1.24 million deaths in India in 2017 attributable to air pollution, 0·67 million were related to ambient particulate matter pollution with another 0.48 million to household air pollution. While the major sources of ambient particulate matter pollution are external, household air pollution is primarily caused by burning of solid fuel for cooking and heating. Chowdhury et al. (2019) estimate that the elimination of biomass for cooking would substantially improve the overall ambient air quality by 17.5%, which has the potential to reduce premature mortality by 6.6%.

While increasing incomes can be expected to reduce the reliance on wood, straw, and cow dung cakes for cooking, and increase the use of cleaner fuels, our calculations based on the IHDS show that the proportion of households using solid fuel, for either cooking or for both cooking and other uses such as heating, barely declined from 76 percent to 73 percent between 2004–05 and 2011–12. Contrasting this mild decrease in solid fuel usage with striking decreases in poverty from 38 percent to 21 percent (Thorat et al., 2017) over the same period suggests that rising incomes may not be sufficient to reduce the reliance on solid fuels. This extensive reliance on biofuels comes at a time when the Government of India has invested in large programs to provide subsidized gas and kerosene for household consumption. Hence, in order to explain the household use of solid cooking fuel, we may need to look at other explanatory factors, particularly the social context in which these decisions are made.

As in most parts of the world, cooking remains a primarily female domain in India. The IHDS finds that in about 98 percent of the households, the primary cook is female. Moreover, among households that rely on wood, the responsibility of collecting it also lies heavily on women and girls (Desai et al., 2010). This indicates the possibility that gender inequalities in Indian society may play an important role in shaping household decisions about investing in clean fuel, and forms the central theme of this paper, which we examine using nationally representative sample from the second wave of the Indian Human Development Survey (IHDS).

The rest of the paper structure is as follows: Section II describes patterns of fuel use in India; Section III discusses the theoretical framework on the need for approaching clean fuel adoption through the gender lens, followed by the hypotheses to be tested; Section IV provides details regarding data, variable definitions, and the methodology; Section V discusses the results; Section VI concludes, with the implications drawn on the basis of our findings.

## 2 Fuel use in India

**Fig. 1:**
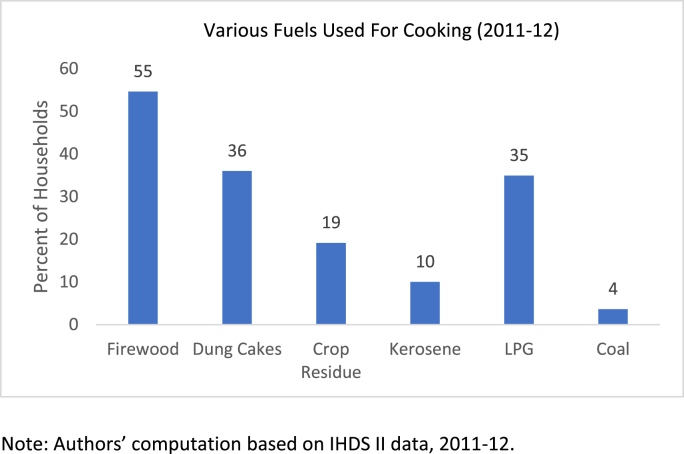
Various Fuels Used For Cooking (2011–12). Note: Authors’ computation based on IHDS II data, 2011–12.

Indian households use a variety of fuels for cooking. Fig. 1, based on IHDS Wave 2, documents the pervasive use of firewood and cow-dung cakes, followed by that of LPG. If we combine the two relatively clean fuels, that is, LPG and kerosene, we find that about 41 percent of the households use these two fuels, though if we focus on households that only use clean fuels, the corresponding figure is barely 22.5 percent. The rest of the households use a combination of kerosene and LPG for some cooking, while retaining the use of firewood and other biomass for items that do not have to be prepared quickly and are required to simmer. For our purpose, we focus on the household use of relatively clean fuels, that is kerosene and LPG for cooking, while eschewing any use of solid fuels for cooking, even at a supplementary level. Among our sample, only 10 percent of the rural households rely solely on clean fuel for cooking, while 51.5 percent do so in urban areas.

While households are slow to adopt the exclusive use of clean fuel, such as kerosene or LPG, for cooking purposes, such adoption is relatively higher across other use-categories depending on the type of fuel. Nearly 50 percent of the households use kerosene for heating and lighting, only 10 percent use kerosene for cooking, another 18 percent for a combination of purposes, including cooking. On, the other hand, 44 percent of the households use LPG for cooking purposes, with 98.4 percent of them using it primarily for cooking.

The Indian government subsidizes both kerosene and LPG cylinders. Kerosene is sold at a subsidized rate to Below the Poverty Line (BPL) households in Fair Price Shops (FPSes) in the neighborhood via the Public Distribution System (PDS) that is governed by central and state governments in the country. However, the unreliable nature of the supply chain distribution system of the PDS network often compels households to opt for biomass and coal, instead of purchasing clean energy from the market at a higher replacement price (Rao, 2012).

Similar to kerosene, access to LPG is also dependent on LPG distribution networks across the country. Further, the high upfront cost and subsequent high refill cost (even after accounting for subsidies) often act as deterrents for households to use LPG as their primary choice of cooking fuel, particularly among the poorest.

**Fig. 2:**
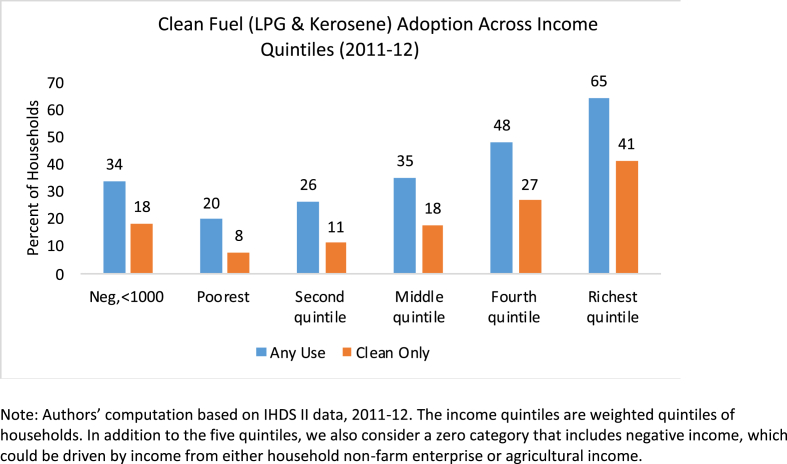
Clean Fuel (LPG & Kerosene) Adoption Across Income Quintiles (2011–12). Note: Authors’ computation based on IHDS II data, 2011–12. The income quintiles are weighted quintiles of households. In addition to the five quintiles, we also consider a zero category that includes negative income, which could be driven by income from either household non-farm enterprise or agricultural income.

The energy ladder hypothesis states that households move toward more expensive and cleaner fuels with increases in income (Muller and Yan, 2018). However, we observe that reliance on biomass for cooking is not limited to the poorest households (see Fig. 2). . While clean fuel usage rises with income, among the richest 20 percent of the Indian households, 65 percent use clean fuels, with only 41 percent relying solely on clean fuel, supporting a weaker form of the hypothesis.

**Fig. 3:**
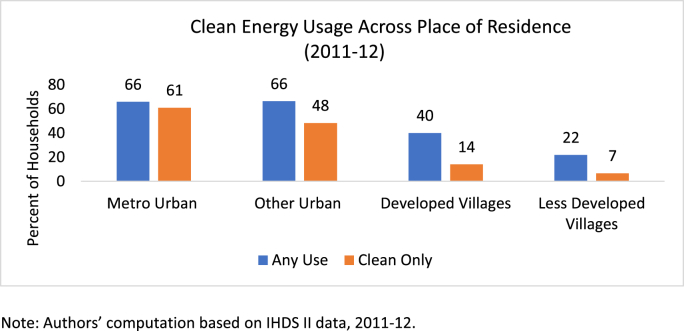
Clean Energy Usage Across Place of Residence (2011–12) Note: Authors’ computation based on IHDS II data, 2011–12.

There is considerable geographical variation in fuel use (see Table 1). While about 30 percent of the households use only clean fuel (Column II of Table 1) for cooking in the northern and southern states, the use of clean fuel is far more limited in the north–central states such as Bihar and Uttar Pradesh. Urban-rural differences are also large, with rural areas lagging behind the peri-urban or metropolitan areas in terms of clean energy adoption (see Fig. 3). These can be driven by access to roads and infrastructural facilities, institutional structures, and distribution networks, apart from local socio-economic considerations.

**Table 1:** Distribution of clean fuel use by state (2011–12).

	Percent of Households	
	Any use	Only Clean
	I	II
Jammu & Kashmir	81	36
Himachal Pradesh	68	37
Uttarakhand	46	25
Punjab	61	30
Haryana	64	23
Delhi	96	87
Uttar Pradesh	24	11
Bihar	24	8
Jharkhand	24	8
Rajasthan	44	14
Chhattisgarh	19	9
Madhya Pradesh	28	16
Northeast	63	46
Assam	41	17
West Bengal	36	17
Orissa	20	8
Gujarat	55	37
Maharashtra & Goa	41	27
Andhra Pradesh	53	33
Karnataka	43	39
Kerala	84	21
Tamil Nadu	51	35
All India	41	22

Note: Authors’ computation based on IHDS II data, 2011–12.

The reliance of households on biofuel tends to increase if they have greater access to cost-free alternate sources of biofuel such as wood or cow-dung, either because of their proximity to common property resource, such as an open access forest area, or if they own agricultural farm land or livestock, which are typically observed in rural areas. Data show that 54 percent of the households that own or cultivate land continue to use bio-fuel for cooking; with 55 percent for households that own livestock.

While such differences in trends in clean fuel usage makes it imperative for us to view clean energy adoption in terms of physical access, along with financial considerations of the household, such aggregate analysis at the household level suffers from the notion that clean fuel adoption is not guided by intra-household dynamics. Our paper puts particular emphasis on intra-household dynamics, focusing on gender-based inequality as a determinant of household fuel choice, as women are primary end-user of cooking fuel, and thus most affected by such choices, but often lack the power to enforce clean fuel use. Using the gender lens, our goal is to examine ways in which women’s access to rights and resources can induce greater adoption of clean fuel for households across all income quintiles.

## 3 Literature review and theoretical framework

### 3.1 Rationale for including gender in research on fuel choice

As the discourse in economics began to move from the perception of households as being ruled by a benign dictator (Becker, 1993) to ones in which different household members negotiate a variety of myriad decisions that shape daily lives (Folbre, 1986), a substantial body of literature has begun to emerge that tries to identify gender differences in preferences and in resources that improve the bargaining power of women vis-à-vis men (Woolley, 1993; Seiz, 1995). Women’s access to income and voice have been identified as factors that enhance their negotiating power in the household domain (Agarwal, 1997; Kabeer, 1994, Kabeer, 1999).

If we wish to argue that women’s increased agency results in changes in consumption behavior, we must first establish that men and women have different consumption and expenditure preferences. Only limited literature exists linking differing gender preferences in fuel choice (Gould and Urpelainen, 2019), but in other domains of life, evidence suggests that men and women do not pool their resources within the household nor do they have similar expenditure preferences across a range of cultures (Alderman et al., 1995; Chiappori, 1997). Evidence from sub-Saharan Africa suggests that women’s access to resources, in the form of income, land, or assets results in increased share of food in household budget (Doss, 2006) and greater dietary diversity (Duflo and Udry, 2004). In Bangladesh, women’s empowerment has been linked to higher per adult-equivalent calorie availability and dietary diversity (Sraboni et al., 2014). Further, when it comes to investing in children, mothers seem to have a greater preference for child-specific investment than fathers,^1^ particularly investments in daughters (Thomas, 1994; Menon, Van Der Meulen Rodgers, and Nguyen, 2014) with evidence supporting improvements in human capital (Holvoet, 2004; Doepke and Tertilt, 2011), nutrition, and health (Shroff et al., 2009; Bose, 2011).

Prima facie, it seems logical that women would have greater preference for investing in cleaner fuel. Using data from two districts in Himachal Pradesh (Parikh, 2011) noted that the arduous task of collecting, transporting and processing biomass has adverse health effects, including injuries and accidents. With women primarily responsible for carrying out such tasks, they remain more prone to such ill effects. However, whether conditions that empower women in making other decisions result in higher investments in clean fuels, remains an empirical question. We would expect that since women are the ones who are most inconvenienced by having to cook in a smoke-filled environment, to collect firewood, and to make cow dung cakes, they would be most motivated to invest in clean fuel. But, studies have also noted a greater preference for collective welfare among women (Croson and Gneezy, 2009) than among men, and women may well neglect their own welfare in preference for investments in other consumption goods that benefit the whole household. Hence, in this paper, using data from the multi-topic IHDS, we examine whether an improvement in conditions leading to women’s higher ability to implement their preferences actually results in the greater use of clean fuels. Past literature in this area is limited in geographic scale and conceptualization of women’s autonomy. For example, Gould and Urpelainen (2019) show that households where women are the primary decision maker are more likely to adopt LPG for cooking. However, this study is limited to six states in the rural areas of north and west India, ignoring tremendous heterogeneity in gender relations across India, and focuses on a single explanatory variable, household decision making in purchase of durable goods. In contrast, our analysis extends to both rural and urban India across all 34 states and union territories (barring Andaman and Nicobar and Lakshadweep). Additionally, we adopt a multi-dimension approach to gender empowerment, with a focus on economic, familial, and cultural factors leading to higher autonomy (Kabeer, 1999; Malhotra et al., 2002) that could potentially influence household fuel choice.

### 3.2 Processes linking gender hierarchies and fuel choice

We argue that gender-based inequalities influence household fuel choices in the two ways: the first changes the value of women’s time, the second changes intra-household power dynamics.

(1) Women have historically borne a greater share of unpaid work such as care-giving services, household chores, and free collection of goods and services (Agarwal, 1997; England, 2005). While caring for children or homes generates benefits that are not easy to substitute, there are few inherent benefits to undertaking what has come to be defined as “domestic drudgery”, as women try to save money by collecting firewood in an effort to save expenditure (Agarwal, 1986). One would expect households to purchase fuel if it increases the time available to women to participate in wage work.
(2) Since the time and health burden of biofuels is disproportionately borne by women, in households where women have greater bargaining power, it will skew household consumption towards items that benefit women’s health and convenience, namely clean fuel. . A careful review by Köhlin et al. (2011) in the 2012 World Development Report on Gender Equality and Development notes that access to clean fuel improves the quality of life for women, but cautions on policy-level targeting without taking into account local energy alternatives, gender roles, and the household decision-making process.

Building on a wide range of literature on different dimensions of gender inequality (King and Mason, 2001; Malhotra et al., 2002) and the unequal access to resources in the household (Kanbur and Haddad, 1994; Duflo and Udry, 2004), we examine the household-level energy decision by focusing on three different dimensions of women’s empowerment. These dimensions capture economic factors, intra-household factors, and cultural factors, respectively. Our focus on three different dimensions of gender inequality is inspired by the fact that gender inequalities exist in a variety of domains, and each domain does not reflect that same underlying construct (Mason, 1995; Balk, 1997; Narayan and Deepa, 2006). Factors that affect intra-household decisions may not affect cultural norms regarding publicly visible activities (Desai and Temsah, 2014) and hence, it is important to focus on various theoretically motivated aspects of gender inequality differently. Our measures of women’s empowerment are as follows:

a) *Economic: Employment Status* measured by whether women participate in paid work;
b) *Family: Decision-makin*g measured by women’s control over household decisions; and
c) *Cultural: Mobility* measured by women’s ability to travel alone when they go outside the home.

### 3.3 Hypotheses

Below we describe the rational and literature associated with our focus on the three dimensions of gender inequalities and articulate hypotheses we test in this paper:

1. *Economic:* In keeping with the available literature (Agarwal, 1997; Sen, 1990), we argue that women’s ability to obtain paid work and their participation in it increases the value of women’s time and health, and increases their control over household decisions to invest in clean fuel. As women move towards paid employment, the likelihood of switching to clean fuel increases – this can be the result of pure income effect via addition to household income, or substitution effect by reducing the time available for domestic work. While unpaid work on the family farm also contributes to the overall household resources, it is the visible work in regular paid labor that is likely to bring the greatest benefits (Anderson and Eswaran, 2009). Thus, we focus on women’s participation in either a salaried job that involves regular monthly income or engagement in a small business.^2^ The data show that around 15% or ever married women are engaged in salaried or business work in urban area, while the corresponding number is 7.5% in rural areas. We, thus, test the following hypothesis:

H1 Women’s employment in salaried work or non-farm business will be associated with higher use of clean fuel for their households.

2. *Family:* If men and women have different preferences (Folbre, 1994; Kanbur and Haddad, 1994; Agarwal, 1997) when it comes to fuel choice, we would expect that women who have greater authority in making household decisions may have a greater ability to convert this authority into choosing clean fuels. There is some evidence that households in which women have greater decision-making power are more likely to invest in improved cooking stove (Mohapatra and Simon, 2017) and adoption of LPG (Gould and Urpelainen, 2019).

H2 An increase in decision-making authority for women will result in greater household use of clean fuel.

3. *Mobility:* In many communities, women suffer from restricted mobility (Jejeebhoy and Sathar, 2001). Social and cultural norms may restrict women’s ability to move freely or travel alone, which in turn, can impair women’s agency by affecting their human capital development, economic empowerment, or access to goods and services, including household fuel choice. The IHDS data show that irrespective of whether they need permission or not, approximately 67 percent of Indian women can travel alone to a health center, while 48 percent reported travelling alone on a bus trip. We focus on whether women can go alone to certain places—these include travelling to a health center, a friend or relative’s house, a *kirana* shop (local small business grocery shop), and a bus trip. We expect that women who have greater physical mobility may find it easier acquire goods and services, and will be more likely to purchase kerosene from fair price shops and may be more likely to ensure replacement of LPG gas cylinder. The three hypotheses have also been summarized in Box 1
  H3 Women who have greater physical mobility will be more likely to acquire clean fuels for cooking.
  Box 1 **Hypotheses and Operationalization**
  **Table:** 

Hypotheses	Operationalization of Key Independent Variable	Hypothesized Direction of Effect on use of clean fuel
Women’s employment in salaried work or non-farm business will be associated with higher use of clean fuel for their households.	Employment defined as respondent’s participation in work where she received monthly salary or was self-employed. She must have worked at least 240 h in this activity during the preceding 12 months.	+
An increase in decision-making authority for women will result in greater household use of clean fuel.	Decision making authority codede Cas 1 if the respondent has most say over any one of the decisions on whether to purchase expensive items, buy land, or how much to spend for social functions.	+
Women who have greater physical mobility will be more likely to acquire clean fuels for cooking.	Physical mobility coded as 1 if respondent was able to go alone to at least one of the listed places (grocery store, health clinic, home of a friend or relative in the neighborhood, a short distance by bus or train)	+
  Alt-text: Box 1

## 4 Data and methodology

### 4.1 Data: India Human Development survey (IHDS)

India Human Development Survey (IHDS), which is the source of data for this paper, included face-to-face interviews with respondents from 41,554 households in 2004–05. About 83 percent of these households were re-interviewed in 2011–12, which also included split households from the original sample, as long as they also resided within the same community. The sample size in Wave 2, conducted in 2011–12, consists of 42,152 households located across 33 (now 34) States and Union Territories (UTs) of India, excluding only the UTs of Andaman & Nicobar Islands, and Lakshadweep Islands. This includes the 83 percent of the 2004-05 households that were reinterviewed, along with all the split households and a refresher sample. The urban sample was refreshed by adding 2114 households in order to address greater rate of attrition in urban areas. The IHDS is a multi-topic survey, containing information about fuel use in addition to data on household income, consumption, employment, and various markers of gender relations in the household. The interviews included administration of an income/employment questionnaire (typically responded to by the male head of the household) and a household energy/gender/health questionnaire (responded to by an ever-married woman).^3^ We focus on the extent to which women’s control over income and their role in household decisions shape household choice regarding the use of clean fuel for cooking. The paper does not seek to establish causality; we examine how alternate markers of women’s empowerment are associated with clean energy adoption.

The household energy use module in IHDS includes detailed questions about the use of firewood/twigs, dung cake, crop residue, kerosene, LPG, and coal. For each fuel, the respondent is asked whether that type of fuel is used for cooking, heating, lighting, or a combination of purposes. This allows us to create an outcome variable, Clean Fuel Use ($CF_{i}),$ that is one if the household is using kerosene or LPG for cooking and does not use any other source of cooking fuel such as firewood or cow-dung cakes, and zero otherwise. Many Indian households use gas or kerosene for making quickly-needed items such as tea, but may rely on a wood burning stove for slow simmering items like lentils or smoked items such as meat. We define use of clean fuel as one in which kerosene or LPG is used for cooking and no other source.

The survey also included a battery of gender-related questions. For our purpose, we focus on three sets of items: (1) women’s employment in paid work; (2) Women’s control over household decisions such as what to cook, whether to buy an expensive item, how many children to have, and what to do when she falls sick, among other things; for each of these items, we ask who participates in the decisions, and when multiple people participate, who has the most say; and, (3) Whether women have the freedom to go alone to nearby places such as a grocery shop, friends/neighbors’ homes, and health clinics. The sample used for this paper is restricted to households that were also administered the “eligible woman’s” questionnaire, where eligible woman is defined as an ever-married woman aged between 15 and 49 years. As of 2011–12, the IHDS provides data for 35,344 eligible women. This restriction is necessary because the questions about decision making and mobility, which allows us to examine household energy adoption in relation to women’s empowerment, are asked only of the ever-married women ages 15–49 years. For a subset of the analysis, where we examine changes over time, we restrict the analysis to 21,243 women, who were interviewed during both waves of the survey.

### 4.2 Variable definitions and operationalization

#### 4.2.1 Dependent variable

The dependent variable, $CF_{i},$ is a binary variable that indicates whether the household belonging to eligible woman *i* uses only clean fuel for cooking.*.*

#### 4.2.2 Key independent variables

We focus on three measures of women’s autonomy ( $X_{i})$:

(1) Employment status defined as 1 if the respondent worked for at least 240 h in the preceding 12 months in business or in a salaried job. Note, that our focus here is on jobs that have some level of stability around which expenditures can be planned. Work as a casual laborer at daily wages is excluded, but salaried work where the respondent is paid on a monthly basis is included.
(2) Decision-making variable measures whether the eligible woman has the most say in any of the expenditure decisions, viz., – decisions on whether to purchase expensive items, buy land, or how much to spend for social functions. We construct a dichotomous variable that takes the value of one when the eligible woman has the “most say” in any one or more of these three categories, and zero if they do not wield decision-making authority over any of these three categories. Our data show that only 17 percent of ever-married women have the deciding voice over any one of the expenditure categories that we consider for our analysis.^4^
(3) Mobility is measured using a variable which takes the value of one if a woman can travel alone to any of the following places: health center, friends’ place, *kirana* (grocery) shop, or a short bus trip, and zero if she does not. Among our sample, 85 percent of ever-married women enjoy the freedom to travel alone to any of these places, irrespective of whether they need permission or not.^5^

Table 2a provides a summary statistic of key variables used in this paper, including control variables, for households that use clean energy only for cooking and ones that do not. Apart from alternate measures of women’s empowerment, we also control for other factors that can potentially influence household fuel choice. These include characteristics of the ever-married woman in the household, such as her age and education. Past literature show that women’s education enhances her ability to negotiate adoption of modern energy and technologies (Lewis and Pattanayak, 2012), and, in general, improves her intra-household bargaining power (Agarwal, 1997). We further include household-level characteristics, such as the number of adult female in the household, whether the household owns agricultural farm, or livestock, whether the household has electricity, whether they are below poverty line (BPL) ration card holders, caste and social group they may belong to, the place of residence, annual household income, and state fixed effects.

**Table 2A:** Summary statistics.

Percent of Households		
	Clean Fuel Usage (LPG/Kerosene)	
	0	1
Age:		
≤ 29 years (Omitted)	81.05	18.95
30–39 years	76.28	23.72
40–49 years	75.25	24.75
Education		
Illiterate (Omitted)	90.27	9.73
Primary	81.81	18.19
Middle	73.57	26.43
Secondary	56.68	43.32
*Women’s Autonomy Indicator*		
Employment in salaried job or business	65.54	34.46
Mobility (travel alone: any one of the four)	77.4	22.6
Decision over purchase (any one of three)	77.5	22.46
Owns farm	89.04	10.96
Owns livestock	91.92	8.08
Electricity	73.7	26.3
BPL ration card holders	82.88	17.12
Social Groups		
Upper Caste (Omitted)	64.4	35.6
OBC	78.42	21.58
Scheduled Caste	84.73	15.27
Scheduled Tribes	91.84	8.16
Muslim	76.11	23.89
Christians, Jain, Sikh	66.41	33.59
Place of residence:		
Metropolitan City (Delhi, Mumbai, Kolkata, Chennai, Bengaluru, Hyderabad)	38.99	61.01
Other Urban	51.64	48.36
More developed village	85.98	14.02
Less developed village	93.28	6.72

Mean:		
Number of adult female in HH	1.66	1.77
Annual Household Income	107,128	202,150
Unearned income (excluding the woman respondent’s earnings)	99,690	186,244
Sample size (unweighted)	(26,380)	(8954)

Note: Authors’ computation based on IHDS II data, 2011–12. Observations have been weighted using eligible women weights to reflect the 2011 Indian population.

**Table 2B:** Asset ownership (2011–12).

Asset Ownership	Percent of Households (2011–12)
Clean only Fuel (LPG/Kerosene)	25.34
Own Refrigerator	24.2
Own Vehicle (bike or car)	31.56
Own air cooler/air conditioner	17.57
Own television	64.6

Note: Authors’ computation based on IHDS II data, 2011–12. Observations have been weighted using eligible women weights to reflect the 2011 Indian population.

In an alternate specification, instead of using total household income, we consider women’s unearned income in order to examine whether the effect of women’s own earnings on household fuel choice has differential effect from her total unearned household income. While past research has primarily considered total household income as a correlate for clean energy adoption (Lewis and Pattanayak, 2012), we examine the role of intra-household distribution of income, emphasizing on the women’s access to income generating activities. Table 2b presents the summary statistics of household asset ownership for refrigerators, air-coolers or air-conditioners, vehicles, and television sets.

Definitions for all variables, including control variables, are included in Table 1 in the Appendix.

### 4.3 Methodology

In our results section, we first present the descriptive statistics for the key variables using data from the 2011–12 wave of the IHDS. Next, we estimate a binary logit regression model predicting household clean fuel adoption (Equation (1)) as follows:

(1) $$CF_{i}=\;log{\left(\frac{\text{Pr}(clean\;fuel=1)}{1-\text{Pr}(clean\;fuel=1)}\right)}_{i}=\mu_{i}+\;\beta_{1}X_{i}+\;\beta_{2}Z_{i},$$

where “*i*” denotes eligible woman. $CF_{i}$ represents the dependent variable, use of clean fuel $X_{i},$ represents the three key independent variables representing women’s autonomy; while $Z_{i}$represents a set of additional explanatory variables, controlling for individual-level characteristics of the ever-married woman, household level characteristics, region, and state fixed effects. $\mu_{i}$ and $\beta_{\text{i}}$ are parameters to be estimated.

We control for known correlates of women’s autonomy such as age, education, caste/religion, region, household structure, and place of residence identified in prior literature. However, autonomy may be affected by many unobserved factors that are not easy to control and fuel choice may also affect autonomy instead of the reverse. For example, access to clean fuel may reduce work burden increasing ability to participate in the labor market instead of the other way around, or women who have closer relationships with their husbands may have greater power in the household bargaining, as well as an ability to express their preferences in household choices regarding consumption. Distinguishing between autonomy and fuel choice is not easy in this case. We address this challenge via several robustness checks described below.

We carry out three separate analyses as follows:

1. In the first scenario, we consider the entire sample of 35,334 ever-married women in the second wave of IHDS, aged 15–49 years. This is the basic individual-level regression where fuel choice is the dependent variable, and the independent variables comprise the three indicators of gender equality and a set of control variables.
2. Further, we carry two the following sets of robustness checks:
  a. We exploit the panel structure of our data to control for unobserved factors by carrying out an analysis with the lagged dependent variable as one of the co-variates. The analysis for this sample is restricted to women aged 15–49 years, who were administered questions about gender and were interviewed in both 2004–05 and 2011–12. In this case, the sample comprises of 21,243 ever-married women from both urban and rural areas. The matched panel data allows us to look at the effect of income growth on adoption of clean fuel, in addition to the other factors that we take into account in the first scenario.
  b. In a second check, we examine the impact of gender equality on household decisions to invest in clean fuel against its impact on items that may be of interest to all household members, men and women alike, as well as items that are of greater interest to men. This allows us to better isolate gender relevance of expenditure decisions. The gender-neutral items include air conditioners/coolers, refrigerators and televisions. Items that are of greater relevance to men include ownership of motor cycle or a car.

## 5 Results

### 5.1 Clean fuel adoption in 2011–12

Table 3 provides the population-averaged marginal effects from our regression models using data on eligible women’s households from the second wave of IHDS. First, we briefly discuss the general correlates of fuel use and then focus on our key explanatory variable, that is, gender equality.

**Table 3:** Average Marginal Effect for Clean Fuel Usage for Cooking using alternate measures of Women’s Autonomy (2011–12).

Fuel (clean only)	Salaried or Business		Decision (any expenses)	Independent Mobility (any)
	I	II	III	IV
**Autonomy Indicator**	0.0259∗∗∗	0.0136∗∗	0.0185∗∗∗	−0.00688
	−0.0072	−0.00684	−0.00708	−0.00687
Annual Household Income (log)∗	0.0303∗∗∗	0.0346∗∗∗	0.0354∗∗∗	0.0348∗∗∗
[unearned only for column I]	−0.00281	−0.00304	−0.00304	−0.00303
Age: 30–39 years	0.0344∗∗∗	0.0319∗∗∗	0.0314∗∗∗	0.0336∗∗∗
	−0.00596	−0.00595	−0.00582	−0.00591
Age: 40–49 years	0.0532∗∗∗	0.0518∗∗∗	0.0496∗∗∗	0.0536∗∗∗
Ref: Age ≤ 29 years	−0.00632	−0.00632	−0.00632	−0.00631
Education: primary	0.0501∗∗∗	0.0510∗∗∗	0.0512∗∗∗	0.0512∗∗∗
	−0.00706	−0.00704	−0.00703	−0.00703
Education: middle	0.0970∗∗∗	0.0971∗∗∗	0.0971∗∗∗	0.0971∗∗∗
	−0.0079	−0.00787	−0.00784	−0.00787
Education: secondary	0.139∗∗∗	0.139∗∗∗	0.140∗∗∗	0.140∗∗∗
Ref: (1) Ed: illiterate	−0.00816	−0.00809	−0.00808	−0.00808
Owns farm	−0.0165∗∗	−0.0175∗∗	−0.0176∗∗	−0.0180∗∗
	−0.00818	−0.00817	−0.00808	−0.00815
Owns livestock	−0.0881∗∗∗	−0.0892∗∗∗	−0.0893∗∗∗	−0.0899∗∗∗
	−0.00767	−0.00765	−0.0077	−0.00768
Electricity	0.151∗∗∗	0.152∗∗∗	0.152∗∗∗	0.152∗∗∗
	−0.0129	−0.0128	−0.0128	−0.0128
BPL ration card	−0.0287∗∗∗	−0.0283∗∗∗	−0.0281∗∗∗	−0.0279∗∗∗
Ref: (0) APL	−0.00564	−0.00562	−0.00562	−0.00564
Other Backward Classes (OBC)	−0.0301∗∗∗	−0.0296∗∗∗	−0.0296∗∗∗	−0.0294∗∗∗
	−0.00715	−0.00713	−0.0071	−0.00711
Scheduled Caste	−0.0739∗∗∗	−0.0738∗∗∗	−0.0740∗∗∗	−0.0735∗∗∗
	−0.00722	−0.00719	−0.00719	−0.00719
Scheduled Tribes	−0.0951∗∗∗	−0.0954∗∗∗	−0.0953∗∗∗	−0.0950∗∗∗
	−0.0104	−0.0103	−0.0103	−0.0103
Muslim	−0.0209∗∗	−0.0189∗∗	−0.0190∗∗	−0.0196∗∗
	−0.00834	−0.00835	−0.00835	−0.00835
Christians, Jain, Sikh	−0.0489∗∗∗	−0.0486∗∗∗	−0.0481∗∗∗	−0.0480∗∗∗
Ref: Upper Caste	−0.0138	−0.0137	−0.0137	−0.0137
Number of adult female in HH (log)	0.00132	0.000121	0.000722	−0.00026
	−0.00573	−0.00574	−0.00573	−0.00572
Other Urban	0.013	0.014	0.0134	0.0145
	−0.0112	−0.0111	−0.0111	−0.0112
More developed village	−0.149∗∗∗	−0.149∗∗∗	−0.149∗∗∗	−0.149∗∗∗
	−0.0127	−0.0125	−0.0125	−0.0126
Less developed village	−0.180∗∗∗	−0.180∗∗∗	−0.180∗∗∗	−0.180∗∗∗
Ref: Metro Urban 1	−0.0128	−0.0127	−0.0126	−0.0127
State Fixed Effects	Yes	Yes	Yes	Yes
Observations	34,226	34,473	34,473	34,473
Wald chi2 (42)	5030.28	5092.89	5030.28	5030.28

***Notes:*** (a) Authors’ computation based on IHDS II data, 2011–12. Coefficients reflect population-averaged marginal effect (probability) from logistic regression for each specification. All results use delta-method standard errors in parentheses, with ∗∗∗p < 0.01, ∗∗p < 0.05, ∗p < 0.1. Observations have been weighted using eligible women weights to reflect the 2011 Indian population.

(b) Column I uses eligible women’s unearned income, whereas columns II, III, and IV use total household income belonging to eligible woman’s household.

*Control Variables:* In Table 3, age, education, and income are all monotonically related to the choice to use clean fuel. Older women, women with more education, and women from higher income families are more likely to live in households that use clean fuel. These coefficients do not change substantially as we include different markers of gender equality one by one. Ownership of a farm or livestock is negatively related to clean fuel use since these households have easy access to crop residue or cow-dung. Households with access to electricity are also more likely to use clean fuel. There are substantial caste and religious differences in clean fuel use, with upper-caste Hindus most likely to use clean fuel and members of the Scheduled Tribes (STs) least likely to use clean fuel. The incidence of lower use of clean fuel among Christians, Jains, and Sikhs is a little surprising, but this relationship may be dominated by Sikhs who tend to own large farms. Small sample sizes do not allow us to further disaggregate this data. Residents of metropolitan cities and other urban areas are the most likely to use clean fuel, while individuals living in less developed villages are the least likely to use clean fuel. Controlling for current income, poor households, that were identified by the government as being poor vide the issuance of a BPL card, are less likely to use clean fuel. This may reflect chronic poverty that transcends current income.

Table 3 also indicates that the eligible woman’s age is positively associated with a higher likelihood of adoption of clean energy fuel, with households comprising ever-married women aged 40–49 years more likely to adopt clean fuel than those with women aged 30–39 years in comparison to households with ever-married women aged 20–29 years. The results are robust across all regressions using alternate measures of women’s autonomy. This shows that women’s intra-household decision-making power also increases with age, which is consistent with the age effects of ever-married women, as noted in prior literature. We also observe the linear effect of adoption of clean energy with women’s education. As compared to women who are not literate, which is our reference category, education has a positive and statistically significant relationship with clean fuel usage, with higher levels of education among the ever-married women increasing the likelihood of the household using only clean fuel for cooking. The prevalence of a higher number of ever-married woman in the household is also vital, as can be seen from the results, as this could lend greater collective voice to women in household-level decision-making.

1. *Women’s Employment in Salaried Jobs or Business:* The first two columns of Table 3 include women’s employment in salaried work or business as an independent variable. The two models differ in that one controls for the total annual income (column II) of the total households (which includes women’s earned income) whereas the second model includes only household income while excluding women’s earned income (column I). The results show that households in which women earn independent incomes are more likely to use clean fuel than households in which women do not, and this increase is positive and statistically significant (at a one percent level). This confirms our first hypothesis, H1.

An increase in log of total household income is associated with increases in probability of clean fuel adoption by 3.5 percentage points (column II). We find similar effect if instead we consider an increase in the woman’s unearned income (column I), with difference in 43 basis points.^6^ While, the difference between the two measures of income is negligible, we observe that disentangling household income from women’s earning potential increases the probability of clean fuel adoption from women’s engagement in business or salaried work. The increase in probability from women’s employment leading to the adoption of LPG or kerosene is higher at 2.6 percentage points when we separate women’s earnings from the total annual household income (see Column I of Table 3 where we control for unearned income) as compared to increases in probability by 1.4 percentage point in the specification where we use the total annual income (column II, Table 3). This indicates that the marginal benefit from women’s earnings is not the same as the marginal benefit of an extra rupee from the general household income, confirming hypothesis H1.

2. *Women’s Control over Expenditure Decisions:* The third column of Table 3 suggests that women’s control over economic decision-making (refer Hypothesis H2) is positively associated with clean fuel usage. The probability of clean fuel usage increase by 2 percentage point when an ever-married woman has decision-making authority over large purchases, such as household expenditure on expensive items, or land, or social functions (see column III of Table 3). This also confirms our hypothesis H2 on the autonomy effect of the ever-married woman in the household, and is also consistent with findings from Gould and Urpelainen (2019), which shows an increase in increase in the probability of LPG adoption of 3.5 percentage points when the woman is the primary decision maker for purchase of durable goods. We also find similar results when we consider a decision-making index that counts the number of decisions over which an ever-married woman has the most say—the higher the decision-making authority, the greater is the likelihood of clean fuel usage. Our results, however, do not hold, if we consider decision-making over traditional women-centric issues such as what to cook. Such roles compartmentalize women and men into separate spheres, where women take responsibility over household chores, while men are responsible for sources of livelihood and budgetary allocations.
3. *Women’s Ability to Travel Alone to Any of the Designated Places*: Model 3 (column IV) includes the impact of women’s independent mobility. It is very small and not statistically significant, implying a lack of relationship between women’s mobility and household fuel choice. The direction of the relationship is negative. This may indicate that if women are less mobile, then the household is more likely to adopt clean fuel, as using other forms of biofuel may induce women to venture out, especially if women are primarily responsible for collecting or sourcing cooking fuel. In the case of communities that more deeply adhere to the gendered notions of space, private or public, mobility can be inversely associated with clean fuel usage. While this refutes our Hypothesis (H3), the results are not statistically significant. We also use another alternate measure of mobility that counts the number of scenarios wherein the woman can travel alone to any one of the referred places—the results are still not significant and robust to this alternate specification.

### 5.2 Robustness checks

#### 5.2.1 Check 1: Panel analysis to control for endogeneity

Although women’s employment or control over expenditure decisions may influence household fuel choices, it is also possible that the causality works in the opposite direction. The use of clean fuel may increase the time available for women to participate in wage work. In order to examine the role of this endogeneity, we carry out a robustness check, with a matched panel data, using both waves of IHDS for both urban and rural areas. While this reduces our sample size to 21,243 ever-married women, the key advantage of using a matched panel of ever-married women is that it allows us to control for the lagged dependent variable, that is, the household adoption of clean fuel for cooking in 2004–05, for all our model specifications, apart from examining the effect of growth in annual household income over the two time periods. We control for income from the base period, 2004–05, as the initial reference income for the household belonging to the ever-married woman and add a variable measuring income growth. For our specification using women’s employment status as an autonomy indicator, we replace household income for women’s unearned income for 2004–05 and the growth of unearned income between the two waves.

Controlling for the lagged dependent variable, in addition to the other explanatory variables discussed so far, allows us to factor in the adoption of clean fuel by the household from a prior period, which could be governed by a range of factors such as the individual-level ever-married women’s autonomy status in 2004–05, or the household’s standard of living, or other factors, during the concerned time period. Our results, presented in Table 5, suggest that the autonomy status of ever-married women, in terms of decision-making, continues to be positively associated with increased likelihood of household level adoption of clean fuel, and is statistically significant at the 5 percent level, while mobility is negatively associated and not significant at conventional levels of the p-value. Women’s employment status in salaried jobs or business is still positively linked to clean fuel usage. However, the variable is significant now only at the 10 percent level, after accounting for unearned household income in 2004–05, and growth in unearned income over the two periods.

**Table 4:** Clean fuel usage for cooking for matched ever-married Women’s panel.

Fuel (clean only)	Salaried or Business	Decision (any expenses)	Mobility (any)
	I	II	III
Autonomy Indicator	0.0155∗	0.0257∗∗	−0.00087
	−0.0089	−0.0129	−0.0107
Annual Household Income 2004–05 (log)∗	0.0336∗∗∗	0.0389∗∗∗	0.0377∗∗∗
[unearned only for column I]	−0.00562	−0.00628	−0.00609
Income Growth between waves I & II	0.0206∗∗∗	0.0280∗∗∗	0.0273∗∗∗
[unearned only for column I]	−0.00421	−0.00452	−0.00453
State Fixed Effects	Yes	Yes	Yes
Observations	20,345	20,726	20,726
Wald chi2 (43)	2993.83	3133.33	3078.06

***Notes***: (a) Authors’ computation based on IHDS waves I and II data, (2004–05 and 2011–12). Coefficients reflect population-averaged marginal effect (probability) from logistic regression for each specification. All results use delta-method standard errors in parentheses, with ∗∗∗p < 0.01, ∗∗p < 0.05, ∗p < 0.1. Observations have been weighted using eligible women weights to reflect the 2004–05 population. The table shows only key indicators. All specifications for Table 4 control for lagged (2004–05) dependent variable, i.e. household adoption of clean only fuel for cooking. Full results are available on request.

(b) Column I uses eligible women’s unearned income, whereas columns II, and III use total household income belonging to eligible woman’s household.

**Table 5:** Effect of gender indicators on household-level asset ownership (2011–12).

Asset Ownership (dependent variable)	Salaried or Business	Decision (any expenses)	Mobility (any)
	I	III	II
Clean Fuel Use (From Table 3)	0.0259∗∗∗	0.0185∗∗∗	−0.00688
	−0.0072	−0.00708	−0.00687
Refrigerator Ownership	0.0239∗∗∗	0.000301	−0.0140∗∗
	−0.0073	−0.00662	−0.00686
Vehicle	0.0263∗∗	−0.0395∗∗∗	0.961∗∗∗
	−0.0104	−0.00784	−0.00754
Cooler/Air-conditioner ownership	0.0055	0.00576	−0.0116∗
	−0.00668	−0.00675	−0.00619
Television (colored or black and white)	0.0430∗∗∗	−0.0031	−0.0186∗∗
	−0.00963	−0.00745	−0.00866

***Notes***: (a) Authors’ computation based on IHDS waves II data (2011–12). Coefficients reflect average marginal effect with identical logistic regressions for the four dependent variables. All results use delta-method standard errors in parentheses, with ∗∗∗p < 0.01, ∗∗p < 0.05, ∗p < 0.1. Observations have been weighted using eligible women weights to reflect the 2011 Indian population. The table shows only estimates for various measures of women’s autonomy from each of the twelve regression specification. Various explanatory variables that have been controlled for are as described in Table 3. Full results are available on request.

(b) Column I uses eligible women’s unearned income, whereas columns II, and III use total household income belonging to eligible woman’s household.

#### 5.2.2 Check 2: Comparison of decisions about clean fuel use with other consumption items

The analysis so far delved into whether the adoption of clean fuel for cooking by households is governed by gender equality. This focus on fuel choice is located in our assumption that since women are both collectors of biofuel and primary cooks, women’s autonomy will have an impact on household choices about investment in clean fuel. However, whether this analysis taps into the gendered nature of household decision-making or reflects unobserved, between household differences is not clear. In order to rule out this possibility, we estimate similar models as those presented in Table 3 for four other outcomes. We argue that while cooking fuel almost exclusively affects women, all household members, including men, women, and children enjoy chilled beverages in a hot climate, cool air, and access to entertainment. In contrast, access to vehicles mostly benefits men, particularly since only about 4 percent of the vehicle owners have a car, while the rest own a bicycle or motorcycle that few *sari*-clad women drive. The question as to whether women’s agency also permeates into other arenas, which are more gender-neutral or male-centric in nature, is perhaps a more rigorous test of women’s intra-household bargaining power.

The comparison of the coefficients for these five assets, as presented in Table 5, is instructive. Women’s participation in wage work seems to contribute to all the ownership of consumption goods suggesting that women’s income increases overall household disposable income and results in overall improvement in standards of living. In contrast, women’s control over the expenditure decision is positively associated with only one item—fuel use. For the rest, it is either not significant or negatively significant (as is the case with the ownership of a vehicle). This bolsters the argument that women may have greater preference for investing in clean fuel. Women’s lack of mobility, which is not a significant determinant of fuel use, seems to be negatively related to the ownership of other goods. This suggests that constraints on women’s physical mobility may have less to do with gender equality than with social class (Balk (1997) comes to a similar conclusion for Bangladesh).

## 6 Conclusion and implication

In this paper, we have shown that households in which women have greater empowerment in the arena of market participation and control over expenditure decisions appear to be more likely to invest in the usage of clean cooking fuels. These results seem to be robust to controls for potential endogeneity. Moreover, these markers seem to have a greater impact on fuel use than items such as the purchase of television sets and coolers that are of interest to all household members.

These results point to the importance of incorporating gender into our considerations as we think about ways of encouraging households to move away from the use of solid fuels to that of clean fuels. Income plays an important role in these decisions as seen by the consistently strong and positive impact of various markers of income in all model specifications. However, income from women needs to be separated from that of the total annual household income, women’s earned income can influence household adoption of clean fuel via both income and substitution effect. Increases in women’s earnings adds to total household income, making available greater share of disposable funds for clean energy adoption through pure income effect. However, evidence also suggests that households at the top quintiles continue to use alternate sources of solid fuel, and this is often governed by local energy markets, or taste and preferences. The result also indicate that when women are engaged in relatively stable and independent work such as salaried jobs or business, households seem to be more inclined to invest scarce resources in clean fuels due to substitution effect, which will free up their time from collecting fuelwood or other biofuel and cooking for long hours, and reduce the negative health impacts for women who are the primary cooks. As women bear greater share of the burden of household chores, such as collecting fuelwood or cooking and cleaning, policies that enhance women’s agency by providing pathways for enhancing skill development and providing employment opportunities are more likely to aid in shifting towards clean fuel adoption. Our results also suggest that women seem to value clean fuels far more highly than other household goods, and when women have greater control over household expenditure decisions, these are reflected in greater investment in clean energy by the household.

Public policies that increase women’s access to independent resources and control over these resources may increase use of clean fuels. Public policies in India and other developing countries (e.g. Progresa in Mexico) have begun to experiment with directing resources to women directly rather than to the household in general. The results presented in this paper suggest that these policies may have substantial environmental and health spillover effects.

## Funding

Funding for the India Human Development Survey Waves 1 and 2 was provided by three grants from the 10.13039/100000002National Institutes of Health (R01HD041455, R01HD0461048, R01HD046166). Supplementary funding for data collection was provided by the 10.13039/100004421World Bank and the 10.13039/100000010Ford Foundation. Analyses presented in this paper have been supported by grants from 10.13039/100000865Bill & Melinda Gates Foundation OPP1183495 & OPP1187930 and from the 10.13039/100004439William and Flora Hewlett Foundation #2018–7924.

## CRediT authorship contribution statement

**Pallavi Choudhuri:** Conceptualization, Data curation, Formal analysis, Methodology, Software, Validation, Visualization, Writing - original draft, Writing - review & editing. **Sonalde Desai:** Conceptualization, Data curation, Funding acquisition, Investigation, Methodology, Project administration, Resources, Software, Supervision, Validation, Visualization, Writing - original draft, Writing - review & editing.

## Declaration of competing interest

The authors declare that they have no known competing financial interests or personal relationships that could have appeared to influence the work reported in this paper.

## Figures and Tables

**Table 1:** Variable Definitions

Variable	Definition
Clean Fuel	1 if the household uses only kerosene or LPG for cooking, it does not use any other energy source
Salaried Work or Business	1 if the respondent participated in work where she received monthly salary or was self-employed. She worked at least 240 h in this activity. 0 if not employed, worked on family farm, or in casual wage labor
Decision-making	1 if the respondent has most say over decisions on whether to purchase expensive items, buy land, or how much to spend for social functions. 0 if she does not wield decision making authority over any of these three categories.
Mobility	1 if respondent was able to go alone to at least one of the listed places (grocery store, health clinic, home of a friend or relative in the neighborhood, a short distance by bus or train) 0 if she cannot go alone to any of these 4 places.
Education of ever-married woman (a set of dummy variables)	Reference category: illiterate Attended primary school Attended middle school Attended secondary school or college
Age of ever-married woman	0 if age ≤ 29 Age 30-39 Age 40-49
Household income	Log of household’s income from all sources
Household income excluding women’s earned income	Log of household’s income from all sources that excludes women’s own wage earnings and women’s share of business income.
Farm household	1 if household owns or cultivates land 0 otherwise
Livestock ownership	1 if household has any livestock including cows, goats, camels and chicken 0 otherwise
Caste and Religion	Reference category: Upper caste, including Brahmins Other Backward Classes (OBC or middle castes) Schedule Caste Scheduled Tribe Muslim Christian, Jain and Sikh
Poor Household	1 if household was identified as a Below Poverty Line (BPL) household and given a BPL card 0 otherwise
Number of adult women in the household	Log of number of adult women in the household
Place of residence	Reference category: Metropolitan City Other urban areas Develop villages with high levels of infrastructure Less developed villages
State of residence	Control for 22 states with smaller states or union territories combined with adjoining states

**Table 2:** Abbreviation List

Abbreviation	Full Form
BPL	Below Poverty Line
CF	Clean Fuel
FPS	Fair Price Shop
GBD	Global Burden of Disease Studies
IHDS	Indian Human Development Survey
PDS	Public Distribution System
PM2.5	Particulate Matter
UT	Union Territories

## Footnotes

1 See Strauss, Mwabu, and Beegle (2000) for a literature review on intra-household bargaining and mother’s preferences towards child-specific investments.

3 For the survey description, see https://www.ihds.umd.edu/home.The IHDS data from waves I and II are available for download from the ICPSR website: https://www.icpsr.umich.edu/icpsrweb/DSDR/studies/37382. A constructed file with just the variables used in this paper is available from authors upon request.

6 100 basis point = 1 percentage point.

## Footnotes

2 The alternative classification of women’s employment status is available upon request. These results suggest that when it comes to predicting fuel choice, non-employment, employment in the family farm, or casual wage employment have similar impacts.

4 In an alternate specification, we also consider a decision-making index with each decision contributing a score to a continuous index with similar results.

5 In an alternate specification, we construct a mobility index that adds up each location where the respondent can travel alone to create a continuous variable. There results with this index are similar to the results from the binary variable we have included.
